# Supplementary material for: Biological Evaluation of Valeriana Extracts from Argentina with Potent Cholinesterase Inhibition for the Treatment of Neurodegenerative Disorders and Their Comorbidities—The Case of Valeriana carnosa Sm. (Caprifoliaceae) Studied in Mice
Source: Pharmaceuticals (Basel). 2023 Jan 16;16(1):129. doi: 10.3390/ph16010129 (PMC9861714; doi:10.3390/ph16010129)
Supplement: Supplementary file 1 [file pharmaceuticals-16-00129-s001.zip › pharmaceuticals-2136364-supplementary.pdf]

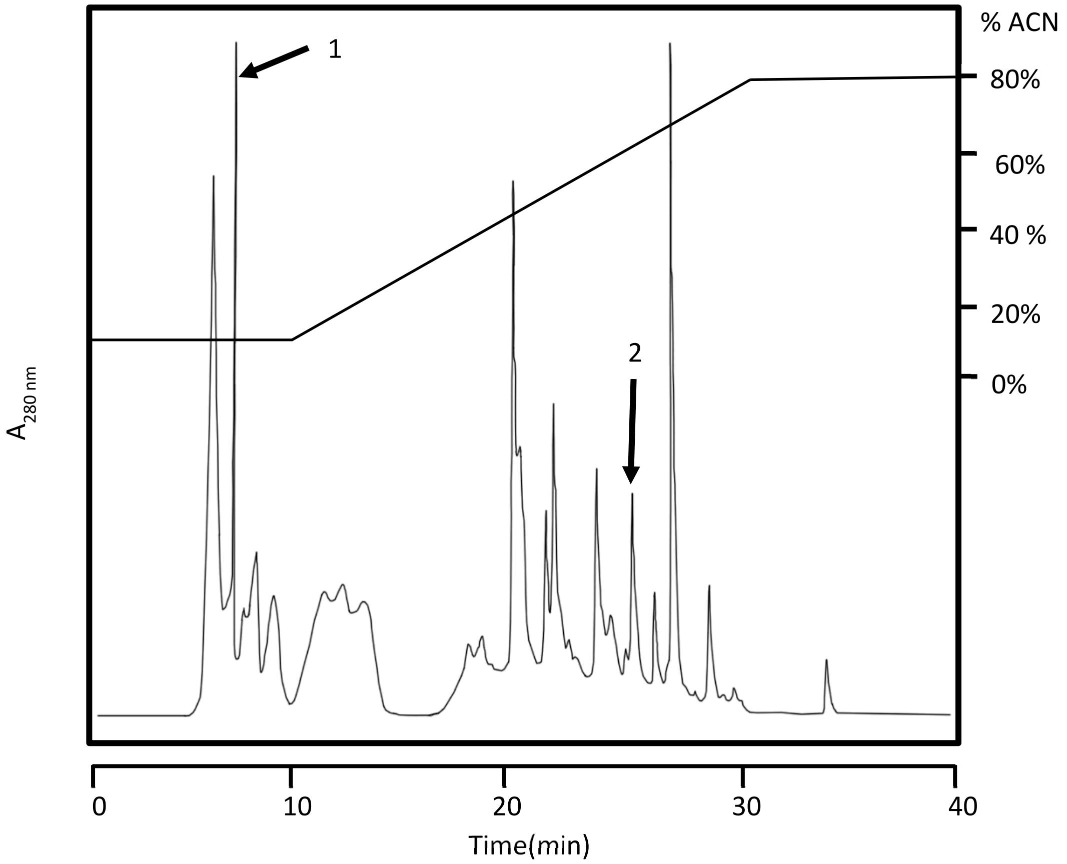

Figure S1: Representative analytical HPLC chromatogram of *V. carnosa* aqueous 1 extract. Fractionation was performed on an LKB Pharmacia instrument using a C-18 reversed-phase Vydac column. The extract was properly injected into the column and eluted with an aqueous/ACN gradient, as shown in the figure at a flow rate of 1 ml/min. Detection was performed at λ = 280 nm. The numbered arrows indicate the retention times of chlorogenic acid (**1**) and 2*S*(-) hesperidin (**2**).

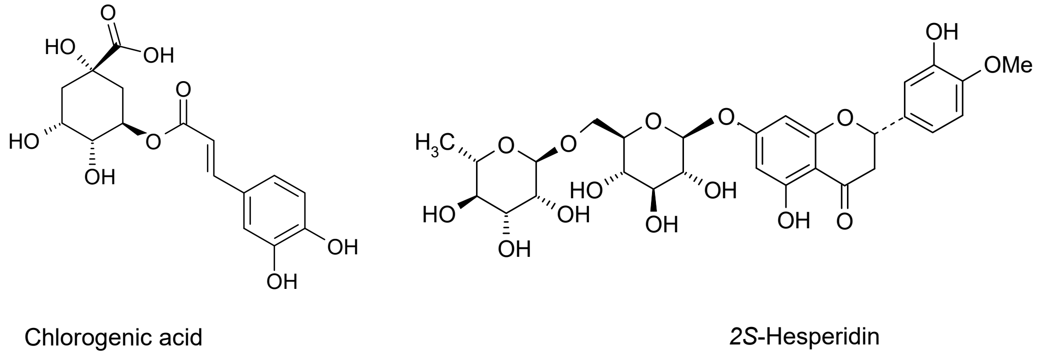

Scheme S1: Chemical structures of chlorogenic acid and 2*S*-hesperidin.

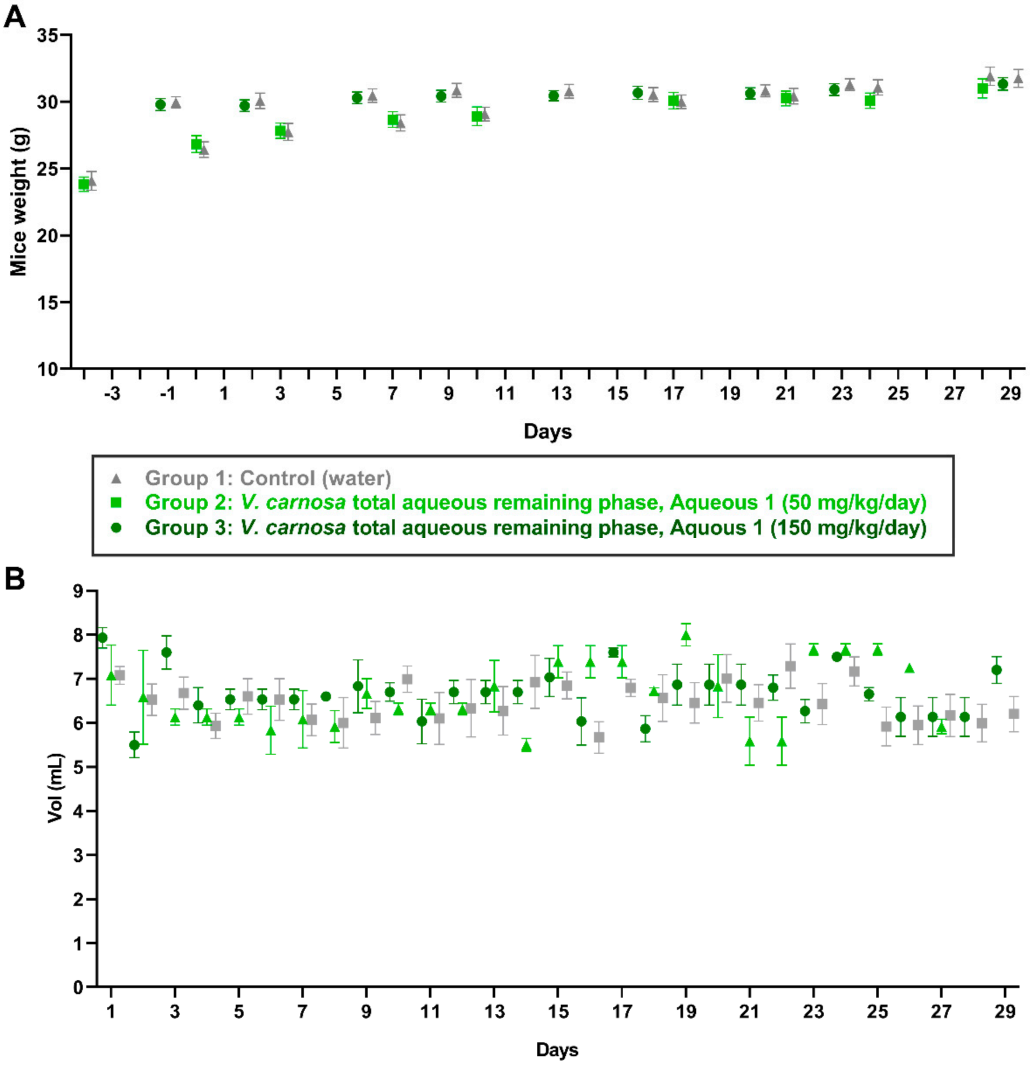

Figure S2: A) Mouse weight and B) volume of fluid intake/day/mice during chronic treatment with *V. carnosa* aqueous 1 extract. Each point corresponds to the mean ± SEM. $n_{group\ 1} = 14$, $n_{group\ 2} = 12$, $n_{group\ 3} = 12$.

Table S1. Details of the "Valerians" (family *Caprifoliaceae*) used in this study.

Plant materials	Site of collection in Argentina[1] (GPS coordinates)	Date	BAB, BAF[1]
V. carnosa Sm.	Río Negro Province, Departamento Capital Cerro Otto: south hillside (41° 09′ 37′′ S; 71° 22′45′′ W)	16-III-2012	H. G. Bach & F. O. Robbiati 578 (BAB, BAF)
V. clarionifolia Phil.	Río Negro Province, Departamento: Pilcaniyeu: 2–3 Km North West from the National airport runway (41° 09′ 43′′ S; 71° 10′ 05′′ W)	15-III-2012	H. G. Bach & F. O. Robbiati 561 (BAB, BAF)
V. macrorhiza DC.	Neuquen Province: Departamento: Ñorquin, Copahue, Mallin: West to the camping (37° 49′ 0,3′′ S; 71° 06′ 25′′ W)	13-III-2012	H. G. Bach & F. O. Robbiati 540 (BAB, BAF)
V. effusa Griseb.	Córdoba Province. Departamento: Colón, Unquillo, Capilla de Bufo (31° 11′ 31′′ S; 64° 21′47′′ W)	26-XI-2010	H. G. Bach 428 (BAB, BAF)
V. ferax (Griseb.) Höck.	Córdoba Province. Departamento: Punilla, Camino de las altas cumbres, the Condor on the side of the provincial road (31° 37′ 9′′ S; 64° 43′42′′ W)	26-XI-2010	H. G. Bach 460 (BAB, BAF)
V. officinalis L.	San Juan Province. Departamento: Calingasta, Barreal (31° 33′ 48′′ S; 69° 27′ 56′′ W)	III-2010	M. Ferres s/n (BAB 23452)

[1]Reference [10].
